# Variable Rate Irrigation of Maize and Soybean in West-Central Nebraska Under Full and Deficit Irrigation

**DOI:** 10.3389/fdata.2019.00034

**Published:** 2019-09-24

**Authors:** J. Burdette Barker, Sandeep Bhatti, Derek M. Heeren, Christopher M. U. Neale, Daran R. Rudnick

**Affiliations:** ^1^Natural Resources Consulting Engineers, Fort Collins, CO, United States; ^2^Biological Systems Engineering Department, University of Nebraska-Lincoln, Lincoln, NE, United States; ^3^Daugherty Water for Food Global Institute at the University of Nebraska, Lincoln, NE, United States; ^4^West Central Research and Extension Center, University of Nebraska-Lincoln, North Platte, NE, United States

**Keywords:** site-specific irrigation, deficit irrigation, remote sensing, irrigation management, evapotranspiration modeling

## Abstract

Variable rate irrigation (VRI) may improve center pivot irrigation management, including deficit irrigation. A remote-sensing-based evapotranspiration model was implemented with Landsat imagery to manage irrigations for a VRI equipped center pivot irrigated field located in West-Central Nebraska planted to maize in 2017 and soybean in 2018. In 2017, the study included VRI using the model, and uniform irrigation using neutron attenuation for full irrigation with no intended water stress (VRI-Full and Uniform-Full treatments, respectively). In 2018, two deficit irrigation treatments were added (VRI-Deficit and Uniform-Deficit, respectively) and the model was modified in an attempt to reduce water balance drift; model performance was promising, as it was executed unaided by measurements of soil water content throughout the season. VRI prescriptions did not correlate well with available water capacity (*R*^2^ < 0.4); however, they correlated better with modeled ET in 2018 (*R*^2^ = 0. 69, VRI-Full; *R*^2^ = 0.55, VRI-Deficit). No significant differences were observed in total intended gross irrigation depth in 2017 (VRI-Full = 351 mm, Uniform Full = 344). However, in 2018, VRI resulted in lower mean prescribed gross irrigation than the corresponding uniform treatments (VRI-Full = 265 mm, Uniform Full = 282 mm, VRI-Deficit = 234 mm, and Uniform Deficit = 267 mm). Notwithstanding the differences in prescribed irrigation (in 2018), VRI did not affect dry grain yield, with no statistically significant differences being found between any treatments in either year (*F* = 0.03, *p* = 0.87 in 2017; *F* = 0.00, *p* = 0.96 for VRI/Uniform and *F* = 0.01, *p* = 0.93 for Full/Deficit in 2018). Likewise, any reduction in irrigation application apparently did not result in detectable reductions in deep percolation potential or actual evapotranspiration. Additional research is needed to further vet the model as a deficit irrigation management tool. Suggested model improvements include a continuous function for water stress and an optimization routine in computing the basal crop coefficient.

## Introduction

Traditionally, the smallest practical management scale for center pivot irrigation has been the field scale. Thus, irrigation applications are typically intended or assumed to be spatially uniform throughout a field. Variable rate irrigation (VRI) technology for center pivots allows for the management scale to be much smaller and application rates to vary within a VRI equipped field. Consequently, VRI has been the subject of recent research (e.g., Stone et al., 2015; Sui and Yan, 2017). As remote sensing techniques may provide opportunity to quantify spatial variability of irrigation requirements, a number of studies have investigated various remote-sensing-based technologies for VRI management (infrared thermometers, O'Shaughnessy et al., 2015; canopy reflectance, Stone et al., 2016; and Landsat thermal and multispectral imagery, Barker et al., 2018b). In western Nebraska and other areas of the High Plains, irrigation water withdrawal may be limited in availability, or limited by regulation. In some of these areas (and other areas globally), significant soil variability exists, further complicating irrigation management. VRI may be beneficial in such areas as the utility of VRI has been demonstrated for fields with variable soil properties (Hedley and Yule, 2009). The utility of VRI in improving efficiency of irrigation (e.g., reducing the fraction of irrigation that runs off, drains below the root zone or is otherwise not available for extraction by plant roots), e.g., in situations of limited water supply, has also been demonstrated including deficit irrigation management (O'Shaughnessy et al., 2016). One reason that VRI may be of interest in deficit irrigation management is that it may allow for a more spatially uniform managed level of water stress, by accounting for soil, and other variability.

While the benefits of VRI have been studied as cited above, a common misunderstanding regarding new irrigation technologies is that they “conserve” water, with reduced pumping resulting in more water available to downstream users. In an inefficient irrigation system, much of the “inefficiency” is water that deep percolates past the root zone and recharges the aquifer. Irrigation technology, if properly managed, may reduce aquifer withdrawals, and reduce deep percolation, also reducing aquifer recharge but potentially decreasing water quality issues. From a watershed scale perspective, the only way to conserve water in the aquifer is to reduce consumptive use or evapotranspiration (Allen et al., 2003). Stakeholders are rightly concerned about aquifer level declines and the subsequent impacts on streamflow. However, the hypothesis of the current study is that, while VRI can be used to reduce pumping or increase yield, it does not significantly reduce consumptive use. In this study this is explored using a spatial evapotranspiration model to manage VRI.

The Spatial EvapoTranspiration Modeling Interface (SETMI) described by Geli and Neale (2012) has been modified and implemented in VRI research in maize and soybean (common commodity crops in the High Plains) by Barker et al. (2018a) and Barker et al. (2018b). This model includes a reflectance-based crop coefficient evapotranspiration (ET) model, wherein the basal crop coefficient is related to a multispectral vegetation index (Neale et al., 1989). SETMI uses relationships developed for maize and soybean in Nebraska by Campos et al. (2017). SETMI also contains a version of the two-source energy balance (TSEB; Norman et al., 1995) as detailed in Barker et al. (2018a). Notably, SETMI contains what is referred to as a hybrid methodology, wherein ET from an energy balance model is incorporated into the water balance through statistical interpolation (Geli, 2012; Neale et al., 2012; Barker et al., 2018a). This hybrid model is intended to allow the model to compensate for model drift in the soil water balance (Neale et al., 2012; Barker et al., 2018a,b).

While SETMI had been tested for VRI management in western Nebraska (Barker et al., 2018b), it had only been tested on maize and the soil water balance was found to drift compared to neutron attenuation soil water measurements (Barker et al., 2018b). Subsequently, modifications have been made to SETMI discussed by Barker et al. (2018b), including dampening surface soil evaporation and provisions for incorporating soil water measurements into the model. These modifications had not previously been tested, though such are included in concurrent research by Bhatti (2018). Finally, no attempt has previously been made to test SETMI for deficit irrigation in row crops in the western High Plains, though others have studied deficit irrigation in the area, for example, Payero et al. (2005) studied deficit irrigation in soybean in West-Central Nebraska. However, they did not include VRI in their study.

The objective of the present study was to determine whether VRI management using SETMI, when compared to “uniform” irrigation, resulted in reduced irrigation applications, reduced consumptive use (ET), improvements in yield, and/or reduced impact on local water resources as represented by reductions in deep percolation (*DP*) potential. A secondary objective was to determine whether SETMI would perform well as a deficit irrigation management tool, which could be accomplished by accurately simulating soil water content.

## Materials and Methods

SETMI was tested as an irrigation management tool in a 2-year field experiment in maize in 2017 and soybean in 2018 in western Nebraska. This experiment builds upon previous work by Barker et al. (2018b), and is complimentary to research by Bhatti (2018). Primary response variables were yield, change in soil water storage over the measurement period (Δ*SWS*), estimated deep percolation (*DP*) during the measurement period, the sum of the latter two variables (Δ*SWS*+ *DP*), modeled ET (*ET*_*c*_), and water balance measured ET (*ET*_*a*_). The Δ*SWS*+*DP* represents an approximation of the impact on deep drainage from the treatments during the measurement periods (beginning prior to imposing treatments and ending near harvest). It represents potential differences in groundwater recharge, and nutrient leaching capacity.

### Study Location

**Figure 1:**
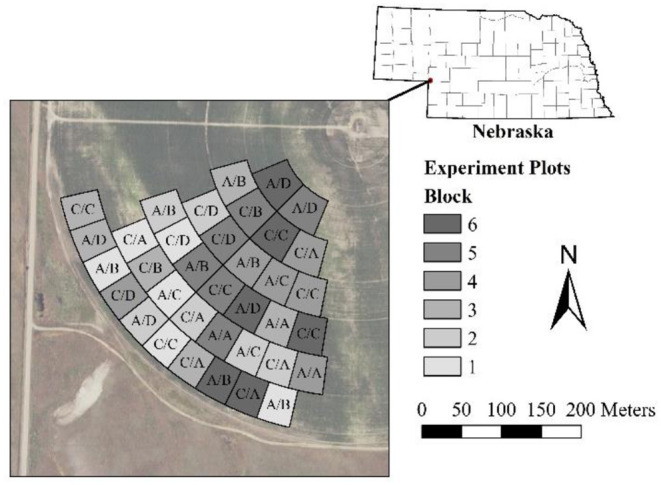
Experimental study area. Letters in plots are treatments (2017/2018). Treatments are: (A) VRI-Full, (B) VRI-Deficit, (C) Uniform-Full, and (D) Uniform-Deficit. Image source: USDA (2016). Political Boundaries sources: USDA (2009a,b).

The study location for this experiment was a quarter of a ~48-ha center pivot irrigated field, which is part of the University of Nebraska-Lincoln's West Central Water Resources Field Laboratory, near Brule, NE (41.027°N, 101.973°W; Google Earth Pro, accessed 8 August 2018; Figure 1). The field is irrigated with a Zimmatic (Lindsay Corporation, Omaha, NE) center pivot equipped with Lindsay Precision VRI with individual nozzle control. The pivot was new in 2015 and is equipped with Senninger XiWob UP3 sprinklers (Senninger Irrigation Inc., Clermont, FL) on drops ~2 m above the ground surface (Barker et al., 2018b).

Soils in the study area range from loam (Santana loam) to loamy sand (Bankard loamy sand) and loam–gravel complex (Santana-Dix complex) (Soil Survey Staff, 2018). The terrain is undulating hills with soil texture varying with topography. The Koppen-Gieger Climate class for the location is Bsk (cold arid, dry summer; Kottek et al., 2006; Brugger, 2017).

Crops were planted following a circular pattern and the center pivot wheel tracks. The field was under continuous maize production for several years prior to rotating into soybean in the 2018 season. The field was managed as no-till, but was subject to a residue removal study prior to 2017 (van Donk et al., 2012; Barker et al., 2018b). In 2017, the study area was under a maize crop Dekalb DKC55-20RIB planted at 78,740 seeds ha^1^ on 8 May 2017. A total of 231 kg ha^−1^ of N was applied with ~25% applied through fertigation. The maize was harvested for grain between 4 and 16 November 2017. In 2018, the crop was soybean Pioneer P24A99X, planted at 457,720 seeds ha^1^ on 8 May 2018. The soybean crop was harvested for grain on 26 September 2018.

### Treatments and Irrigation Management

In 2017, two irrigation treatments were applied: (1) VRI using SETMI with Landsat imagery, and (2) uniform irrigation using neutron attenuation based measurements of soil water content to provide feedback for irrigation scheduling. Both treatments were full irrigation, with no intentional water stress, thus they are referred to as VRI-Full, and Uniform-Full, respectively. In 2018, two additional treatments were added. These two treatments were both deficit irrigation and corresponded to the two full irrigation treatments in the modeling and neutron probe feedback approaches (VRI-Deficit and Uniform-Deficit, respectively). Thus in 2018, the experiment became a two-by-two factorial layout. Deficit irrigation as defined here included an irrigation management allowed soil water depletion that was greater than would be used for minimal water stress management during part of the growing season. Additionally, in 2017, SETMI was executed using the hybrid method and incorporating neutron-attenuation-measured soil water content. However in 2018, SETMI was executed without these two methodologies (i.e., the reflectance-based crop coefficient ET model based on Landsat satellite multispectral inputs and water balance were exclusively used). This was because the hybrid methodology was found to induce model drift necessitating the incorporation of neutron probe measurements. By eliminating the hybrid model in 2018, a SETMI-only approach could be tested. This was deemed more beneficial than maintaining the 2017 modeling approach.

The irrigation was managed similarly to Barker et al. (2018b), using the concept of a managed root zone water balance and management allowed depletion (Woodruff et al., 1972; Martin et al., 1990). This method, used by Barker et al. (2018b) provides a convenient way to manage VRI and to apply treatments to VRI equipped irrigated fields. The basics of the method are (Barker et al., 2018b):

- Determine soil properties (e.g., available water capacity) for each plot or management area.
- Determine a management allowed depletion fraction for the treatment or field.
- Determine a target stored root zone soil water depth above management allowed depletion after irrigation, to which the root zone will be refilled during an irrigation event. A value of ~20 mm (0.8 in) was used in this study thus providing a soil water storage buffer for rainfall capture.
- Compute the water balance, forecasting ET forward in time (precipitation could also be forecasted; soil water measurements could be incorporated). Similar to Barker et al. (2018b), the VRI treatments received plot-specific irrigation based on mean modeled soil water depletion within a ~9 m buffer area in each plot. In this study, the water balance for the VRI plots was computed using SETMI.
- Irrigation application depth is computed for any desired date as the difference between the target water content (the specified depth above management allowed depletion) and the modeled water content at the end of the day in question assuming no water inputs after inputting the available water record. This difference, if positive, is the net irrigation requirement for the plot or management area to be applied by the end of that day.
- Irrigation is triggered such to prevent any plot from exceeding management allowed depletion (described in the following paragraph). Thus, irrigation events occur at the same time for all treatments, but application rates vary by treatment and/or plot.
- Maximum and minimum gross application rates (accounting for application efficiency) are applied as necessary for pivot and pump management. In this study application efficiency (taken here to be the portion of applied irrigation that was infiltrated) was assumed to be 85%.
- Irrigation prescriptions are set to a practical irrigation application depth (optional). In this study, gross applied irrigation was rounded to the nearest ~5 mm (0.2 in).

For the full irrigation treatments, management allowed depletion (MAD) was set to 50%. Late in the 2017 season, the MAD was allowed to reach 60% as in Yonts et al. (2008), but it was maintained at 50% throughout the season for soybean in 2018. The management allowed depletion was set to 60% for the deficit treatments in 2018; this was increased to 75% after reaching the end pod-fill stage following the principles of Kranz and Specht (2012) who suggest avoiding stress during pod-fill. This strategy implicitly seeks to maximize yield with perhaps only modest reductions in applied irrigation, since water stress may affect soybean yield during pod-filling more than other crop stages (Doss et al., 1974; Momen et al., 1979; Cox and Jolliff, 1986; Kranz and Specht, 2012). The soybean crop was near beginning pod-fill when the treatments effectively began to be applied in 2018; thus the deficit irrigated plots may have been insulated from yield loss per Kranz and Specht (2012). However, Doss et al. (1974), reported that stressing soybean in late pod-fill was more consistent at causing a yield reduction than when stress was only earlier in the season.

Irrigation prescriptions were computed about once per week during the treatment periods. In 2017, the treatment period began on 9 August; prior to this time a total of 182 mm of uniform irrigation was applied. In 2018, the treatment period effectively began on 13 August. Prior to that time, all treatments effectively received 119 mm of uniform irrigation.

### Modeling

#### Modeling for Irrigation Scheduling

Irrigation prescriptions for the uniform plots were based upon neutron-attenuation-measured soil water content (see the Measurements and Response Variables section) and a short-term simple soil water balance including ET modeled using crop coefficients based on (Allen and Wright, 2002) for maize in 2017 and using relationships reported by Irmak et al. (2013) for soybean in 2018. Uniform treatments received irrigation based on neutron-attenuation-measured soil water depletion and a simple water balance for one plot of the treatment in 2017 and the maximum irrigation requirement of two selected plots in each of the 2018 uniform treatments.

Irrigation prescriptions for the VRI treatments were computed using the SETMI application, with model formulations similar to Barker et al. (2018a) and Barker et al. (2018b). The SETMI water balance model was modified prior to use in 2018 to include other updates to the FAO56 (Allen et al., 1998) methodology, which are described in Jensen and Allen (2016). The model includes a daily water balance generally following Allen et al. (1998), with ET computed using reflectance based crop coefficients following Campos et al. (2017) and Barker et al. (2018a). In 2017, the hybrid method within SETMI was included using TSEB-computed ET (using the Priestly-Taylor approximation for canopy latent heat flux; Norman et al., 1995; Colaizzi et al., 2014) and a statistical interpolation weighting factor from Barker et al. (2018a) equal to 0.56. To prevent model drift, in 2017, measured soil water depletions from neutron attenuation measurements were incorporated into the model as the actual end-of-day root zone depletion on the measurement date. In this, neutron attenuation measurements from four plots in the VRI-Full treatment were compared to model output depletion for the same plots. The four selected plots had the maximum, minimum, 33 percentile, and 66 percentile values of volumetric water content measurements, respectively, for that treatment on 27 June 2017. The reasoning was to include locations spread across the distribution of soil water content. Modeled depletion for all computation pixels was then updated based upon correlation between these measurements and modeled soil water depletion if *R*^2^ ≥ 0.6, or the mean difference between the measured and modeled values for those four locations otherwise as in Bhatti (2018). This adjustment was to reduce model drift. In 2018, this methodology was not used because excluding the hybrid methodology was expected to reduce model drift. For the 2018 simulations, the model was initiated with soil water content assumed to be at field capacity.

#### Spatial Soil Properties

Prior to execution in 2018, the SETMI water balance model was also modified to include a slow root zone drainage routine using the drainage equations of Raes et al. (2017); soil below the root zone was assumed to have instantaneous drainage whenever water content exceeded field capacity. Furthermore, the model was expanded to allow for the input of properties for three soil layers. The properties for the root zone were computed as depth-weighted averages of layers within the root zone on a given day. Saturated hydraulic conductivity (*K*_*sat*_) for the root zone, a key parameter in the drainage formulas (Raes et al., 2017), was computed throughout the season (as root depth increased) based upon Equation 3.10 of Radcliffe and Šimunek (2010):

(1) $$\begin{array}{r} K_{satr}=\frac{Z_{ri}}{\sum_{j=1}^{3}\frac{{\Delta Z}_{rij}}{K_{satj}}} \end{array}$$

where the subscript *K*_*satr*_ is *K*_*sat*_ in the root zone, *Z*_*r*_ is the root zone depth, Δ*Z*_*r*_ is the thickness of a soil layer within the root zone, with subscripts *i* and *j* being time and soil layer, respectively. This method is for steady-state, saturated flow. However, it provided a practical means of computing *K*_*sat*_ for the root zone and allowed the model to stay within the framework of a simple water balance (Allen et al., 1998; Jensen and Allen, 2016).

Soil properties for each plot were estimated from neutron attenuation, lab measurements, and the gSSURGO (Soil Survey Staff, 2015) database. Field capacity (*FC*) was computed using the maximum neutron attenuation measurement from the 1 June and 27 June 2017 measurements for each depth and plot, using the concept of observational *FC* (Lo et al., 2017). For 2017 irrigation management, plot *FC* was then computed by taking the depth-weighted average of this maximum water content from the soil surface to 1 m. For 2018 irrigation scheduling, the same data were used to compute *FC*; however *FC* was computed for three soil layers (0–0.23, 0.23–0.61, and 0.61–1.22 m) using depth-weighted averages. For the final analysis, *FC* was computed as in 2018 irrigation scheduling with the lowest layer being 0.61–1.0 m and the inclusion of an improved neutron probe calibration for 27 June 2017, which changed some of final plot *FC* values.

**Figure 2:**
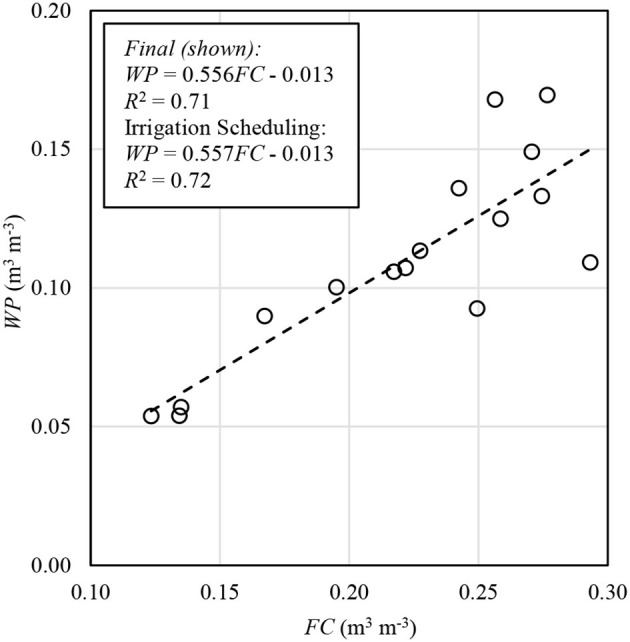
Linear regression fit of permanent wilting point (*WP*) as a function of plot field capacity (*FC*) from 2017 observations.

Permanent wilting point (*WP*) was determined from laboratory measurements on soil samples from 16 plots. *WP* was determined for each sample using a WP4-T Dewpoint PotentiaMeter (Decagon Devices, Pullman, WA) assuming a potential of −1.5 MPa for *WP*. The resulting *WP* values were correlated with respective *FC* values for the same plot and depth from the 1 June and 27 June 2017 data (*R*^2^ = 0.72; Figure 2). The resulting relationship was applied depth-by-depth to compute *WP* for all plots corresponding to the respective *FC* estimation methods (i.e., 2017, 2018, and final analysis). The regression was recalculated for final analysis using the updated neutron probe calibration mentioned above. However, this made no difference in the resulting *WP* for the final analysis when rounded to the nearest 0.01 m^3^ m^−3^.

**Figure 3:**
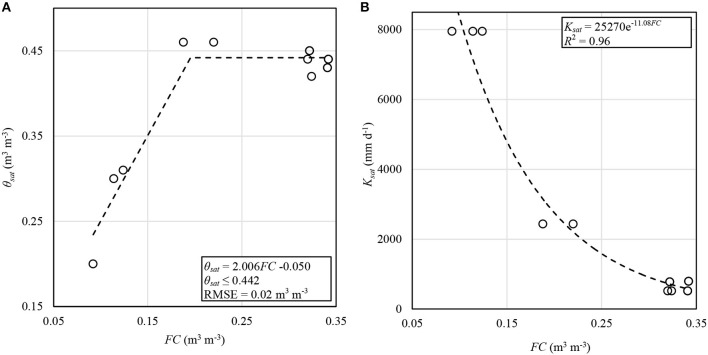
Regression relationships of volumetric soil water content at saturation (θ_*sat*_) **(A)** and saturated hydraulic conductivity (*K*_*sat*_) **(B)** as functions of field capacity (*FC*). All values here were obtained or derived from the gSSURGO database. The piecewise function in **(A)** is a linear regression fit to all data with *FC* < 0.25 m^3^ m^−3^. Fit statistics were computed using the calibration dataset.

In 2018, the inclusion of the drainage equations from Raes et al. (2017) required the input of both volumetric soil water content at saturation (θ_*sat*_) and saturated hydraulic conductivity (*K*_*sat*_). These values were obtained for each plot and soil layer using values from gSSURGO and relationships with *FC*. The *FC* for these relationships was computed based on volumetric water content at −1.5 MPa and available water capacity (*AWC*) for the soil horizons of the dominant components of the four soil map units in the study area. This was done assuming this was a better estimate of *FC* than was water content at −0.033 MPa. A piecewise linear relationship was developed for θ_*sat*_ using linear regression for values of *FC* < 0.25 m^3^ m^−3^ (*R*^2^ = 0.92), and then the average of all θ_*sat*_ for *FC* > 0.25 m^3^ m^−3^ (Figure 3A). A power regression fit well (*R*^2^ = 0.96) for *K*_*sat*_ as a function of *FC* (Figure 3B). The relationships in Figure 3 were used in irrigation scheduling in 2018 and in final analysis, using the respective soil layer *FC* values.

#### Model Input Data

Other spatial model inputs included Landsat 7 and Landsat 8 multispectral imagery obtained from U.S. Geological Survey (https://earthexplorer.usgs.gov/). This imagery included Level 2 Surface Reflectance and Level 1 thermal infrared imagery (2017 only). Thermal infrared imagery were corrected for atmospheric effects similar to Barker et al. (2018a) and Barker et al. (2018b), whose method was based upon Brunsell and Gillies (2002). The correction parameters from NASA's Atmospheric Correction Parameter web app (https://atmcorr.gsfc.nasa.gov/, by J. Barsi) were used. Local surface weather data described below were input to the web application. Only Tier 1 and Real Time imagery were used for irrigation scheduling (with the exception of 5 August 2017, which was a Tier 2 image, included as Real Time quality). In the final analysis, only Tier 1 imagery were included. Landsat 7 imagery were included if no pixels were missing due to the scan line gap over the entire study plot area. Thirteen images were included in 2017 and 14 images in 2018. Not all images were included in irrigation scheduling or final analysis (Table 1); for example, images were included in the final analysis that were not available during irrigation scheduling and some used in irrigation scheduling in 2017 were determined to not be of sufficient quality to use in final analysis. The model was executed using a ground resolution of 1 m in 2017 and 5 m in 2018 and final analysis to account for discrepancies between Landsat pixel size and position and the plot geometry (Bhatti, 2018).

**Table 1:** Landsat images used in the study.

**2017 Maize**					**2018 Soybean**				
**Landsat no**.	Path, Row^*^	**Date**	**Use^†^**		**Landsat no**.	Path, Row^*^	**Date**	**Use^†^**	
7	31, 32	26 May	Iw	Fw	7	32, 32	5 Jun	Iw	Fw
8	32, 32	10 Jun	Iw	Fw	8	31, 32	6 Jun	Iw	Fw
8	32, 32	26 Jun	Iwe	Fw	8	32, 32	13 Jun	Iw	Fw
7	32, 32	4 Jul	Iwe	Fw	7	32, 32	21 Jun	Iw	Fw
8	31, 32	5 Jul	Iwe	Fw	8	32, 32	29 Jun	Iw	Fw
8	31, 32	21 Jul	Iwe	Fw	8	31, 32	8 Jul	Iw	Fw
7	32, 32	5 Aug	Iwe		8	31, 32	24 Jul	Iw	Fw
8	32, 32	13 Aug	Iwe		8	32, 32	31 Jul	Iw	Fw
8	31, 32	22 Aug	Iwe	Fw	8	31, 32	9 Aug	Iw	Fw
8	32, 32	29 Aug	Iwe	Fw	8	32,32	17 Sep		Fw
7	32, 32	6 Sep	Iwe		7	31,32	18 Sep		Fw
8	31, 32	25 Oct		Fw	7	32,32	25 Sep		Fw
8	32, 32	1 Nov		Fw	8	31,32	26 Sep		Fw
					8	32,32	3 Oct		Fw

* *Landsat path and row of the image*.

† *I, used for real-time irrigation scheduling; F, used in final analysis; w, water balance; e, Two-Source Energy Balance*.

Weather data for modeling were obtained from three Nebraska Mesonet (https://mesonet.unl.edu/) weather stations: Big Springs 8NE, Brule 6SW, and Keystone 3W Beta. The Brule 6SW data were used for near real-time data for irrigation management in 2017 and for the final analysis. The Big Springs 8NE data were used for near real-time data in 2018. This weather station was nearby and was used thinking it was the Brule 6SW station, which was adjacent to the research field. The difference was expected to be small; however the Brule 6SW station was still determined to be the best suited for the final analysis. The nearby Keystone 3W Beta data were used to compute 20-year historic average reference ET (*ET*_*r*_) and temperatures for growing degree-day computation, both used for ET forecasting similar to Barker et al. (2018b). This station was used because the records for the Brule 6SW and Big Springs 8NE stations were not sufficiently long. *ET*_*r*_ was computed using the ASCE Standardized Reference ET equation (ASCE, 2005). Barometric pressure was obtained from a nearby cosmic-ray neutron probe station and was provided by T. E. Franz (University of Nebraska-Lincoln). Precipitation from the Brule 6SW weather station was verified in 2017 using a lab-calibrated, Texas Electronics, Inc. (Dallas, TX) TR-525USW rain gauge coupled with a H07-002-04 HOBO Event Data Logger (Onset Computer Corporation, Bourne, MA) installed near the northwest corner of the field. These latter data were also used from 1 to 8 November 2017, because the Brule 6SW dataset did not cover this period. Data from the National Weather Service Global Historic Climate Network Ogallala NE station were used for 9–13 November 2017 because neither the lab-calibrated rain gauge nor the Brule 6SE data were available. Weather data for the Mesonet and National Weather Service stations were obtained from the High Plains Regional Climate Center.

Besides precipitation, the other major water balance input for modeling was applied irrigation. The prescribed irrigation was assumed to be applied (with an application efficiency of 85%—used throughout the study) on the day that the pivot passed over the plots. In the case that the pivot passed over the plots during the night (covering two dates), the irrigation was attributed to plots on the date it was estimated to pass over them in the 2017 irrigation scheduling. The average pivot travel speed from start-stop times and travel angle were used to determine the overpass time for plots. Irrigation depth was assigned typically the latter of the two dates in 2018 scheduling, for simplicity. In final analysis, the earlier of the two dates was used in all cases, because this would make the water available sooner to alleviate any modeled water stress (see the formulations of Jensen and Allen, 2016).

#### Soil Water Content Model Validation

Since the value of SETMI in this research was in modeling soil water content, the model performance was validated using neutron attenuation measurements after the first measurement. Missing measurements in both years and for the 2018 plots that were affected by the irrigation system malfunction during this season were excluded. The model was executed using only the reflectance-based crop coefficient ET and water balance. The first neutron attenuation measurements each year were incorporated into the model as described in the Measurement and Response Variables section.

### Measurements and Response Variables

The total prescribed irrigation depth (the total gross irrigation depth for each plot that was used in the executed irrigation prescriptions) was analyzed as a primary treatment comparison. This was assumed to be equivalent to the actual gross applied irrigation for each plot. The final irrigation in both years (applied 19 September 2017 and 14 September 2018, respectively) were not applied and/or computed as intended. In the case of 19 September 2017, the prescription for the previous irrigation (14 September 2017) was applied in what was probably a logistically practical situation. In the case of 14 September 2018, the applied prescription from 8 September 2018 was not converted from inches to mm before input into SETMI, thus the prescribed irrigation was erroneously high for some plots. In both cases, both total applied prescribed (total prescribed irrigation as defined above) and the total intended prescribed gross irrigation (or the total gross irrigation if correcting for the errors described above in the paragraph) were analyzed. The pivot had some low pressure problems in 2017. However, these may have been related to a pressure sensor (not actual pressure) and were assumed not to have affected sprinkler application and uniformity. To assess the ability of the VRI methodology on accounting for spatially varying irrigation requirements, intended prescribed irrigation was compared to available water capacity (*AWC*) and plot modeled ET (*ET*_*c*_; described later in this section).

Primary measurements included grain yield and soil water content. Plot yield was computed from production yield monitor data, which was cleaned and processed using the U.S. Department of Agriculture Agricultural Research Service's Yield Editor 2.0.7 software (Sudduth et al., 2012) similar to Barker et al. (2018b). Yield points were only included if they were within a ~12 m buffer within each plot boundary (Barker et al., 2018b). Plots included in the final statistics had 22–47 yield points within the buffer in 2017 and 26–45 points in 2018. Dry grain yield was computed using the yield monitor's moisture content measurements.

Soil water content measurements were used in computing the other response variables. Soil volumetric water content was measured in each plot using neutron probes (503 Elite Hydroprobe, CPN, Concord, CA). Access tubes were installed in the approximate center of each plot. The neutron probe instruments used were either locally calibrated or cross-calibrated with a locally-calibrated probe. The local calibration was conducted in a field immediately east of the study site. Local calibration of a gauge used in the study resulted in a slope of 0.2068 and an offset of −0.0607. Calibrations were applied at a 4 decimal accuracy in 2017 and final analysis; they were applied at a higher numerical precision in 2018 irrigation, though this was of little consequence. Neutron attenuation measurements were taken at the following soil depths with measurements assumed to represent the range in parentheses: 0.15 (0–0.23 m), 0.30 (0.23–0.38 m), 0.46 (0.38–0.61 m), 0.76 (0.61–0.91 m), 1.07 (0.91–1.22 m), and 1.37 m (1.22–1.52 m). Neutron attenuation measurements were limited in temporal frequency in 2017 and in seasonal coverage in 2018 (Table 2). However, the coverage in both years began before the treatment period had effectively started and finished near harvest. For modeling purposes in irrigation scheduling, neutron attenuation measurements were treated as the actual root zone water content at the end of the measurement date for the VRI-Full treatment in 2017 and the uniform treatments in both years. However, some neutron attenuation data were shifted to represent the ending conditions on the previous day for final analysis depending on rainfall and irrigation on the measurement day (Table 2). The neutron attenuation data for the 0.91–1.22 m depth for two plots for all 2018 dates except 10 July 2018 were gap filled for final analysis. These were filled using the respective measurement value from the 0.61–0.91 m reading.

**Table 2:** Neutron attenuation measurement dates.

**Maize**	**Soybean**
1 Jun 2017^*^	10 Jul 2018^†^
27 Jun 2017^*^^†^	31 Jul, 1 Aug 2018^‡^
20 Jul 2017	6 Aug 2018^†^
3 Aug 2017	14 Aug 2018^†^
30 Aug 2017	20 Aug 2018
13 Nov 2017	27 Aug 2018^†^
	19 Sep 2018
	25 Sep 2018

* *Used to compute field capacity*.

† *Attributed to previous day during final analysis*.

‡ *All data were used as if collected on 31 July. For irrigation scheduling, only standard neutron counts from 31 July were used*.

The response variables relating to the impact of treatments on *DP* and ET were all derived from the neutron attenuation data. The first was Δ*SWS* in mm in the top 1.0 m of the soil profile (using the 1.07-m reading to represent 0.91–1.0 m) between the first and last neutron attenuation measurement each year (Table 2). Modeled *DP* and *ET*_*c*_ were computed using the SETMI water balance with soil properties described for final analysis. In modeling *DP* and *ET*_*c*_, the depth-weighted neutron attenuation measurements were incorporated as the actual root zone depletions and the average water contents of the soil below the root zone (down to 1.0 m below ground surface) at the end of the first date each year (Table 2). This is similar to the method used by Djaman and Irmak (2013). A similar method, but using repeated soil water content measurements as model input was used by Barker et al. (2018b). ET was also computed using the residual of the water balance (*ET*_*a*_). Both *ET*_*c*_ and *ET*_*a*_ were included as response variables. The final variable considered was the sum Δ*SWS* and *DP* (Δ*SWS*+*DP*). For all of these variables, the neutron-attenuation-measured values were assumed to represent the entire plot area. The plot-average modeled root depth (based upon time to reach peak basal crop coefficient for each pixel) was used to compute root zone depletion and average volumetric water content below the root zone from the neutron attenuation data. The average root depth was computed as a spatial mean for each plot and neutron attenuation date excluding a ~9 m buffer within each plot boundary (Barker et al., 2018b). The model was executed at a 5-m pixel resolution in the final analysis. The response variables were also computed as spatial means excluding the same ~9 m buffer within each plot boundary.

### Experimental Design and Statistical Analysis

The experiment was a generalized randomized complete block design, with multiple replicates of at least some treatments in each block. The study area was broken into plots ~0.2 ha in size accommodating the precision of the sprinkler system and the commercial combine yield monitor similar to Barker et al. (2018b). Plots were arranged radially with plot boundaries falling on pivot wheel tracks (Figure 1). The plots were designed with criteria of a minimum of ~37 m in width and length (Barker et al., 2018b). Plots were grouped into six blocks using computed *AWC* for the top 1 m of soil (as computed for the 2017 irrigation scheduling) as the blocking criterion. Plots were not blocked by radial distance from the center based on results of Barker et al. (2018b). Treatments were randomly assigned to plots afresh in both years (Figure 1).

Responses for each treatment and variable were compared using ANOVAs computed using PROC GLIMMIX in SAS 9.4 (SAS Institute Inc., Cary, NC). The ANOVAs were computed separately for the 2 crop years and for each variable (i.e., no MANOVAs were included). Total prescribed irrigation was compared using 95% confidence intervals computed using SAS PROC MEANS to compare with the uniform treatments as in Barker et al. (2018b). ANOVA was used to compare irrigation for the two VRI treatments in 2018.

A number of plots were discarded from the final analysis based upon missing data or misapplication of irrigation. In 2017, a total of six plots received an erroneous application depth during one of the irrigation events. These plots were all Uniform-Full, the misapplication affected some VRI-Full plots also, but that would have automatically been compensated for in the following irrigation prescription. Another plot had water in the neutron access tube on the 13 November 2017 measurement and was also excluded. All seven plots were excluded from final analysis for 2017. An additional two plots in 2017 did not have neutron attenuation data for 20 July 2017. For 2017, only data for plots and measurement dates that had missing data were excluded from validating the modeled water content. In 2018, two banks of four sprinklers did not function properly during much, if not all, of the irrigation season. Based upon the pivot dealer's sprinkler chart (provided by Holzfaster's Equipment, Ogallala, NE, and computed using software by Senninger Irrigation Inc., Clemont, FL, 6 February 2015), the affected areas were near the center of the second and sixth radial rows of plots from the pivot center (Figure 1). Thus, a total of 14 plots were affected, one of which was also missing neutron attenuation data. All 14 plots were eliminated from the final analysis for 2018. One of the affected plots in 2018 was used to schedule irrigation for the Uniform-Full treatment. However, this treatment always received the maximum irrigation depth during a given irrigation cycle. Given that the affected sprinklers were likely always on (a default conditions of the system; personal communication, Lindsay Corporation personnel), this plot would not have adversely affected that treatment.

## Results and Discussion

### Study Conditions

The May–September total *ET*_*r*_ was 1,020 mm in 2017 and 910 mm in 2018 for the Brule 6SW station, comparable to a 20-years average of 1,080 mm for 1998–2017 for the Keystone 3W Beta station. This is also similar to the 950–1,050 mm reported by Sharma and Irmak (2012) for 1986–2009 for this part of Nebraska. Total May–September precipitation was 280 mm in 2017 and 400 mm in 2018, compared to 290 mm for the 1998–2017 20-year average for Keystone 3W Beta. This is also similar to the 280–320 mm reported by Sharma and Irmak (2012) for 1986–2009 for the area.

In addition to weather, the soil properties at the site may greatly affect an irrigation experiment. Using the final analysis values, *FC* in the top 1 m of the soil profile ranged from 0.11 to 0.30 m^3^ m^−3^. The range of *WP* values was smaller, 0.05–0.16 m^3^ m^−3^. Calculated *AWC* for the plots ranged from 0.06 to 0.14 m^3^ m^−3^. This translates into 60–140 mm over a 1-m managed root zone.

### Modeling and Treatment Execution

**Figure 4:**
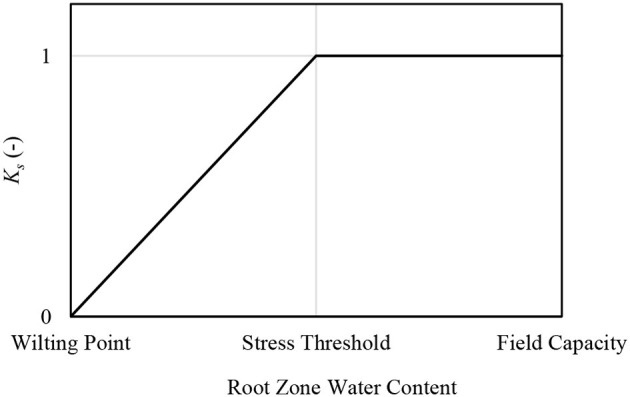
Generalized depiction of the piecewise water stress coefficient (*K*_*s*_) function based on average root zone soil water content, as used in Allen et al. (1998) and Jensen and Allen (2016).

The incorporation of TSEB-ET in 2017 had a general effect of increasing modeled soil water depletion (decreasing soil water content). This was in part because of the nature of the piecewise water stress coefficient used (*K*_*s*_; Allen et al., 1998; Figure 4). That is, *K*_*s*_ is equal to unity when the modeled root zone water content is above the water stress threshold (Allen et al., 1998; Figure 4). In the hybrid methodology, the ET modeled by the reflectance-based crop coefficient method in the water balance (WB-ET) is updated based upon TSEB-ET. If TSEB-ET ≠ WB-ET, then the water balance is updated by back-calculating for *K*_*s*_ and then back calculating for start-of-day root zone depletion (Geli, 2012). For example, assume that the TSEB-ET had little mean bias as compared with the WB-ET. At times, it would be expected that TSEB-ET < WB-ET, even under non-stressed conditions, due solely to model variation. This condition would result in a decrease of *K*_*s*_ after TSEB-ET incorporation, whether such represented reality or not. However, at times when TSEB-ET > WB-ET, and the water balance already indicates a *K*_*s*_ < 1; mathematically, the soil water depletion can only be decreased (soil water content increased) as far as the water stress threshold (Figure 4). It is noted that in cases where *K*_*s*_ = 1 and TSEB-ET > WB-ET, no adjustment is made (Barker et al., 2018a). These challenges led to exclusion of the TSEB-ET in 2018. One possible solution for these challenges may be the inclusion of a continuous *K*_*s*_ function. For example the relationship of Jensen (1970) has been used for similar purposes (Colaizzi et al., 2003, Zhang et al., 2017). Another non-linear relationship presented by Boonyantharokul and Walker (1979) is also promising because the shape could be modified to account for different sensitivity to stress in different crops as is done in the piecewise method used by Allen et al. (1998). A final solution may be incorporation of ET in parts (evaporation and transpiration) using canopy and soil components from the TSEB. This may help better identify what ET differences are truly related to *K*_*s*_. The Penman Monteith formulation of the TSEB has been reported to provide more accurate partitioning of ET than original formulations (Colaizzi et al., 2014).

Possible modifications to the hybrid model aside, the discrepancies in scale of the Landsat thermal infrared imagery (60–100 m; Bhatti, 2018; USGS, 2019) and our plot size was also a reason for dropping the TSEB-ET in 2018. It was evident that incorporating the soil water content measurements into the SETMI water balance in 2017 prevented what seemed to be a likely bias toward a dry modeled root zone. Thus, it was expected that without the TSEB-ET, the neutron attenuation data was less necessary. Furthermore, the water balance model had not previously been tested in this climate for soybean without soil water content measurement incorporation, as it has been for maize (Barker et al., 2018b).

One final challenge encountered with using the SETMI model was the division of Landsat images as being either early or late in the season with regards to computing the basal crop coefficient time series (see Barker et al., 2018a). In this, images either contribute to the increasing portion of the crop coefficient, before and up-to peak value, or they contribute to the decreasing portion of the crop coefficient after peak. In the case of 2017, the best cutoff was not the same for all pixels. For example, if only imagery before 21 July 2017 was included in the development portion of the season, then crop coefficients were poorly fit for ~13 plots. However, if the cutoff was set to include 21 July 2017 in the development portion of the season, all performed reasonably well, but some not as well as if 21 July 2017 was forced to be late in the season. Ultimately, 21 July 2017 was allowed to be a development period image for all plots. The effect was small in terms of modeled root zone depletion. Ultimately, the model would be improved by adding an optimization routine based on goodness of fit to determine the best cutoff date for each pixel.

### Model Performance

**Figure 5:**
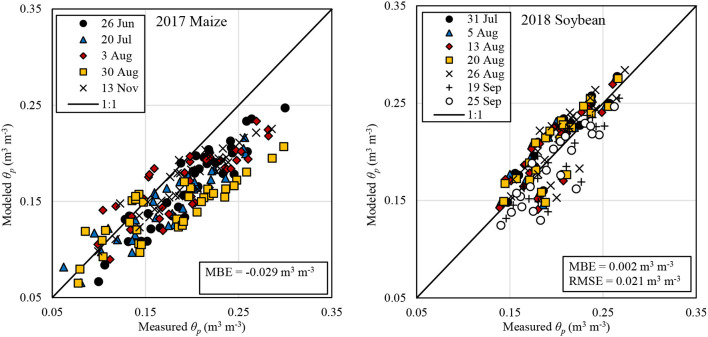
Modeled vs. measured 1-m profile average soil volumetric water content (θ_*p*_) for dates after the first measurement date in 2017 and 2018.

The modeled 1-m average profile soil volumetric water content (θ_*p*_) had a negative mean bias error (MBE) of ~-0.03 m^3^ m^−3^, with a root mean squared error (RMSE) of ~0.04 m^3^ m^−3^, in 2017 (Figure 5). These results may be improved with model calibration, including reducing soil evaporation. Assuming a 1.5-m root zone did not appreciably improve results for 2017. Model performance was improved in 2018 soybean with modeled θ_*p*_ for the top 1-m having a MBE < 0.01 m^3^ m^−3^ and an RMSE ≈ 0.02 m^3^ m^−3^. These results represent an improvement over results in the same field in 2017. Furthermore, these results indicate that the water balance model in SETMI is suitable for irrigation management under these conditions without the periodic incorporation of secondary datasets as was done in 2017. Assessing the uncertainty associated with using SETMI for irrigation management was beyond the scope of this study. However, such is acknowledged as a possible source of variability and error presented in Figure 5.

### Prescribed Irrigation

For the 2017 season, the VRI-Full treatment had a range (across plots) of 314–375 mm for prescribed gross irrigation depth and a range of 304–375 mm for intended prescriptions. When comparing treatment means, the total prescribed irrigation was essentially the same for the VRI-Full and Uniform-Full treatments (353 and 355 mm, respectively; Table 3). The mean total intended prescribed irrigation was also similar (351 and 344 mm for the VRI-Full and Uniform-Full, respectively, Table 3). With a 95% confidence interval of the mean of ± 9 mm for the applied VRI-Full and ± 10 mm for the intended VRI-Full, the two treatments were not statistically different and practically the same. This is logical as both treatments included incorporation of neutron attenuation measurements.

**Table 3:** Mean total prescribed irrigation^*^.

**Treatment**	**2017 Maize**			**2018 Soybean**		
	***n***	**Applied^†^** **(mm)**	**Intended^†^** **(mm)**	***n***	**Applied^†^** **(mm)**	**Intended^†^** **(mm)**
VRI-Full	18	353 [±9]^‡^	351 [±10] ^‡^	6	270 [±3]	265 [±5]^§^
VRI-Deficit	–	–	–	4	239 [±4]	234 [±7]
Uniform-Full	11	355^‡^	344^‡^	6	282	282
Uniform-Deficit	–	–	–	6	267	267^§^

* *Arithmetic means are presented for 2017 and all uniform treatments, least-squares estimated means are presented for the VRI treatments in 2018. Brackets contain 95% confidence intervals. No treatments were significantly different at the α = 0.05 level for either year*.

† *Applied was actually prescribed and executed, Intended includes corrections to the final applied irrigation in each years*.

‡,§ *Pairings not significantly different at the α = 0.05 level as tested with ANOVA (in the case of VRI-Full vs. VRI-Deficit) or 95% confidence intervals of the mean (in comparisons with uniform treatments)*.

In 2018, total intended gross prescribed irrigation application depth ranged from 236 to 277 mm for VRI-Full and 216 to 241 mm for VRI-Deficit. The range was 246–282 mm for applied prescribed gross irrigation for VRI-Full and 221–246 mm for VRI-Deficit. In that year total applied prescribed irrigation was statistically significantly different (*t* = 17.7) for both VRI treatments. Both were also different from the two uniform treatments. Total intended prescribed irrigation was likewise different for the two VRI treatments (*t* = 10.2) with only the VRI-Full and Uniform-Deficit not being different. The greatest total intended irrigation depth was 282 mm for Uniform-Full, followed by 267 mm for Uniform-Deficit, 265 mm for VRI-Full, and 234 mm for VRI-Deficit. Since the uniform treatments had no variability in prescribed irrigation, a factorial analysis was not performed. However, in both cases intended full irrigation was greater than deficit (31 mm greater in the case of VRI and 15 mm greater in the case of Uniform). Likewise, intended VRI was less than Uniform with Full being 17 mm less and Deficit being 33 mm less. It is possible that model bias (Figure 5; which was from the final analysis, but is still illustrative) may have biased the VRI treatments. This could potentially have the impact of increasing prescribed irrigation for the VRI treatments. It is evident that under our study conditions, adoption of VRI based upon reduced water withdrawal alone may not be justifiable, agreeing with the findings of Lo et al. (2016), and Barker et al. (2018b). Justification for VRI would need to include other benefits, e.g., changes in yield, lower cost of production or reduced nutrient leaching (Lo, 2015; Lo et al., 2016). The late imposition of treatments (i.e., the VRI treatments were irrigated similar to the Uniform during the first portion of both years) may also have reduced potential differences. This may particularly be the case for the deficit irrigated soybean treatments in 2019, which may have responded differently had the different treatments been imposed from the beginning of the irrigation season. Furthermore, the Landsat shortwave imagery resolution (30 m; USGS, 2019) may limit the ability to address the soil and other variability within the study field. This may have a smoothing effect that may reduce potential benefits of VRI. Concurrent research is investigating unmanned aircraft systems as a source of high-resolution imagery for VRI management with SETMI (Bhatti, 2018). However, the practical management scale of VRI may be similar in scale to Landsat as discussed by Barker et al. (2018a).

### Irrigation Variability

**Figure 6:**
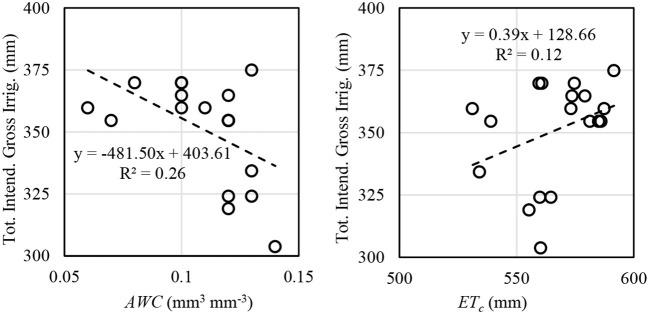
Total gross intended irrigation vs. *AWC* and *ET*_*c*_ for VRI-Full plots included in the final analysis for 2017 maize VRI-Full treatment.

The intent of VRI is to manage irrigation at a sub-field scale to account for sub-field scale heterogeneity. In the framework of the procedure and modeling used in this study, VRI would be anticipated to respond to conditions when spatial variability exists for root zone *AWC* and/or *ET*_*c*_. The total intended prescribed irrigation was compared for these two variables for each VRI plot included in the final analysis. In 2017, the correlation between total intended gross irrigation depth and *AWC* was negative, as expected, but it was poor (*R*^2^ = 0.26, *n* = 18; Figure 6). The poor correlation is likely a result of the hybrid model adjustments to the water balance, which were also responsible for much of the model drift. In 2018, the correlation between total intended gross irrigation depth and *AWC* was similarly poor (*R*^2^ = 0.37, *n* = 6, for VRI-Full; *R*^2^ = 0.10, *n* = 4, for VRI-Deficit). The prescribed irrigation depth would be expected to be highly correlated with *AWC* under conditions of large *AWC* variability in situations where differences in rainfall storage capacity across soils may be mined (e.g., Lo et al., 2016), as may have been expected for 2018, but this was not observed. Scale discrepancies between the SETMI modeled scale, which was dependent upon Landsat imagery resolution (30 m; USGS, 2019) and Landsat pixel positioning, and soil variability may have also contributed. There was a poor positive correlation between intended gross irrigation and *ET*_*c*_ (*R*^2^ = 0.12; Figure 6) in 2017. In 2018 there was a stronger correlation between total intended gross irrigation and *ET*_*c*_ for both VRI treatments (*R*^2^ = 0.69, *n* = 6, for VRI-Full; *R*^2^ = 0.55, *n* = 4, for VRI-Deficit), suggesting that variability in *ET*_*c*_ was a major driver in 2018. Despite the small dataset, the results of the *ET*_*c*_ correlations for 2018 may be better than 2017 because the final *ET*_*c*_ values are close to those used in irrigation scheduling for 2018, but in 2017 irrigation management, the model was updated with other data sources, which may have reduced correlation. Attempts to find correlation between intended gross irrigation and the product and quotient of *ET*_*c*_ and *AWC* generally yielded poor results. The field has variability in *ET*_*c*_ even in the VRI-Full treatment, which is a response derived from the Landsat imagery and soil properties.

### Yield and Soil Water Storage Response

In both years, dry grain yield was similar between all included treatments (*F* = 0.03, *p* = 0.87 in 2017; *F* = 0.00, *p* = 0.96 for VRI/Uniform and *F* = 0.01, *p* = 0.93 for Full/Deficit in 2018). Estimated mean maize grain yield was both statistically and practically similar between treatments with 8.91 Mg ha^−1^ for the VRI-Full in 2017 and 8.98 Mg ha^−1^ for Uniform-Full (Table 4). Variability was, however, notable with 95% confidence intervals of the estimated means being ± 6 and ±8% of the estimated mean for the VRI-Full and Uniform-Full, respectively. In 2018, the dry grain yield was statistically similar between all treatments ranging from 3.33 Mg ha^−1^ for Uniform-Full to 3.10 Mg ha^−1^ for Uniform-Deficit. However, the variability was substantial, ranging from 19% of the estimated mean for Uniform-Full to 24% for the VRI-Deficit. This is also notable in the apparent reversal of expected response for the VRI-Full and VRI-Deficit treatments (3.11 Mg ha-1 and 3.29 Mg ha-1, respectively; Table 4). However, it must be remembered that because of the variance of the data, these values are statistically the same; thus any conjecture about apparent differences is not valid. It is possible that response variance is being induced by the soil variability beyond what is captured in the *AWC* blocking and modeling scale.

**Table 4:** Estimated least-squares means of the response variables^*^.

**Treatment**	**2017 Maize**					
	**Dry Yield** **(Mg ha^−1^)**	***ΔSWS*^†^** **(mm)**	***DP*^†^** **(mm)**	***ΔSWS*+*DP*^†^** **(mm)**	***ET_a_^†^*** **(mm)**	***ET_c_^†^*** **(mm)**
VRI-Full	8.91 [±0.56]	−23 [±8]	0.5 [±0.3]^‡^	−23 [±8]	548 [±9]	567 [±6]
VRI-Deficit	–	–	–	–	–	–
Uniform-Full	8.98 [±0.73]	−20 [±11]	0.1 [±0.3]^‡^	−20 [±11]	544 [±11]	572 [±8]
Uniform-Deficit	–	–	–	–	–	–
**Treatment**	**2018 Soybean**					
	**Dry Yield** **(Mg ha^−1^)**	***ΔSWS*** **(mm)**	***DP*** **(mm)**	***ΔSWS+DP*** **(mm)**	***ET_a_*** **(mm)**	***ET_c_*** **(mm)**
VRI-Full	3.11 [±0.63]	17 [±6]	12 [±11]	29 [±10]^§^	291 [±11]	302 [±5]
VRI-Deficit	3.29 [±0.78]	8 [±8]	5 [±14]	13 [±12]^§^	279 [±14]	300 [±6]
Uniform-Full	3.33 [±0.64]	12 [±7]	22 [±12]	34 [±10]^§^	299 [±11]	303 [±5]
Uniform-Deficit	3.10 [±0.68]	8 [±7]	18 [±12]	26 [±11]^§^	296 [±12]	305 [±5]

* *Means are presented with 95% confidence intervals in brackets. Except as noted, no treatments were significantly different at the α = 0.05 level for either year*.

† *ΔSWS = change in soil water storage in the assumed 1 m root zone; DP = estimated deep percolation from the assumed 1 m root zone. ET_a_ is ET computed from as the residual of the water balance; ET_c_ is SETMI modeled ET. Differences are between November 13 and June 1, 2017 and September 25 and July 9, 2018*.

‡ *Pairings significantly different at the α = 0.05 level*.

§ *Full/Deficit factor main effects different at the α = 0.05 level with Full = 31 mm [±8 mm] and Deficit = 20 mm [±7 mm]*.

Not only did the irrigation treatments not have a significant impact on yield, there were also no significant differences between treatments for the *DP*-related variables (Δ*SWS, DP*, and Δ*SWS*+*DP*; Table 4). In 2017, Δ*SWS* was similar for both treatments (−23 and −20 mm for the VRI-Full and Uniform-Full, respectively). Δ*SWS* is presented as a depth-weighted average down to 1 m for the last neutron attenuation reading minus first neutron attenuation reading (Table 2). When deeper neutron attenuation values were included, down to 1.52 m, the magnitudes increased, but were similar (VRI-Full = −35 mm, Uniform-Full = −36 mm; in these deep estimates, three additional plots were eliminated because of missing deep neutron attenuation readings as compared with the data in Table 4). In 2017, estimated *DP* was likewise similar between treatments and was practically negligible < 1 mm for both treatments, though the difference was determined to be statistically significant.

We acknowledge that the estimates for the *DP*-related variables are dependent upon the water balance; during the period of 2 June−13 November 2017 the total precipitation was 241 mm with an estimated 4 mm of runoff using the NRCS Runoff Equation (NRCS, 2004) with a curve number of 80. In computing *DP*, the SETMI water balance was executed for each plot with the initial soil water content condition on 1 June 2017 taken from neutron attenuation readings. The mean total modeled *ET*_*c*_ between 2 June and 13 November 2017 ranged from 567 to 572 mm for the two treatments. The corresponding *ET*_*a*_ determined from the water balance was 548 mm (VRI-Full) and 544 mm (Uniform-Full), a 19–28 mm difference. This difference could have been reduced by further dampening the surface soil evaporation rate in SETMI or by reducing the water stress threshold or modifying soil properties; however the apparent error was ~5% or less and we deemed these adjustments not justifiable. Interestingly, the differences in applied irrigation did not translate into meaningful differences in ET, *DP*, or Δ*SWS*.

Results for 2018 were similar to 2017, despite the differences in crops, SETMI methodologies, and the inclusion of the deficit treatments. The Δ*SWS* ranged from 8 mm (deficit treatments) to 17 mm for the VRI-Full. Δ*SWS* is presented as a depth weighted average down to 1 m. When deeper neutron attenuation values were included, down to 1.22 m (many measurement tubes were not sufficiently deep for the 1.52 m readings), the magnitudes were practically and statistically similar (5 mm for the deficit treatments, 10 mm for Uniform-Full, and 15 mm for VRI-Full). In 2018, estimated *DP* ranged from 5 mm for VRI-Deficit to 22 mm for Uniform-Full. Significant full/deficit factor main effects were found (*F* = 5.91, *p* = 0.03) for Δ*SWS*+*DP*, with full treatments being 31 mm and deficit being 20 mm (Table 4). Thus, deficit irrigation had a small impact on *DP* potential.

With respect to water balance, the 2018 *DP* estimates were computed for the period of 10 July−25 September 2018. The total precipitation was 125 mm during this period with an estimated 16 mm of runoff (computed as in 2017). In 2018, the *ET*_*a*_ computed as a residual of the water balance ranged from 279 mm (VRI-Deficit) to 299 mm (Uniform-Full), with no significant differences. The SETMI modeled *ET*_*c*_ had less treatment variability ranging from 300 mm (VRI-Deficit) to 305 mm (Uniform-Deficit), again with no significant differences. The net difference between *ET*_*a*_ and *ET*_*c*_ ranged from 5 mm (Uniform-Full) to 21 mm (VRI-Deficit), differences < 8% of *ET*_*a*_. Again, we feel that SETMI performed adequately and that the results did not justify further model adjustments in computing *DP*.

While there was some difference in irrigation in 2018, there was no significant difference in dry grain yield or ET. Interestingly, even the deficit-irrigated treatments where such an effect might be expected, did not result in a significant yield difference. Part of the cause may be that the deficit treatments were effectively not implemented until mid-August (see the Treatments section). However, Doss et al. (1974) found that stressing soybean in late pod-fill was more consistent at causing a yield reduction than when stress occurred only earlier in the season. Moreover, the deficit treatment experienced the greatest managed stress around the end pod-fill stage and later. One final observation is that yield and ET in this field may both be highly governed by nutrient availability in consequence of the light soils (T.H. Lo, UNL, personal communication). Future work should seek to quantify possible nutrient stress. Even though the treatments did not have different ET, there was the 12 mm difference in *DP* potential as represented by Δ*SWS*+*DP* between the full and deficit irrigated treatments. This difference corresponds closely to the difference in applied irrigation between the full and deficit irrigated treatments (12 mm for VRI and 28 mm for uniform). This supports the hypothesis that VRI is unlikely to reduce consumptive use; however it may reduce deep percolation. In stating this, it should be remembered that all treatments were treated similarly in the first portion of both seasons. This may have dampened some of the potential responses.

## Conclusions

Ultimately, the results support the conclusion that, under our study conditions, VRI managed with SETMI resulted in a relatively small, nominal reduction in water withdrawn in 2018 as compared to uniform treatments; however no difference was observed in 2017. These results are subject to model performance, scale, and treatment implementation. Furthermore, irrigation treatments did not result in differences in grain yield. It is evident that any variability accounted for in the VRI management did not result in corresponding detectable changes in yield. Likewise, few statistically significant differences were found in estimated deep percolation, modeled or water balance measured evapotranspiration, change in soil water storage in the top 1 m of the profile, or the sum of these latter two variables between any treatments in either year. More testing should be performed to demonstrate the utility of SETMI for deficit irrigation management. The performance of the SETMI water balance model in 2018 was promising, as it was executed unaided by on-site measurements beyond weather data, *FC*, and *WP*. Testing over longer periods and varied conditions is necessary to fully test that utility. The temporal and spatial scale of Landsat data was a concern (especially thermal imagery); alternate satellite data products and/or unmanned aircraft imagery may address this problem. The proposed modifications to SETMI, including optimization of the basal crop coefficient development, inclusion of a non-linear *K*_*s*_ function, and partitioning TSEB-ET, may also improve model performance.

## Data Availability Statement

All datasets generated for this study are included in the manuscript/supplementary files.

## Author Contributions

JB, DH, CN, SB, and DR contributed to the conception and design of the study. JB and SB performed the modeling for irrigation management, field research, data collection, and data analysis. JB wrote the first draft of the manuscript. SB, DH, CN, and DR reviewed and made contributions to the manuscript. All authors approved the submitted version.

### Conflict of Interest

JB was employed by the University of Nebraska-Lincoln at the time of study, but has since moved to Natural Resources Consulting Engineers, Fort Collins, CO. The remaining authors declare that the research was conducted in the absence of any commercial or financial relationships that could be construed as a potential conflict of interest.
